# Effects of ankylosing spondylitis on cardiovascular disease: aMendelian randomization study

**DOI:** 10.3389/fgene.2024.1359829

**Published:** 2024-06-26

**Authors:** Lu Xiao, Shudian Lin, Feng Zhan

**Affiliations:** ^1^ Department of Rheumatology, The Fifth People’s Hospital of Wuxi, Affiliated Wuxi Fifth Hospital of Jiangnan University, Wuxi, China; ^2^ Department of Rheumatology, Hainan General Hospital, Hainan Affiliated Hospital of Hainan Medical University, Haikou, China

**Keywords:** ankylosing spondylitis, cardiovascular disease, Mendelian randomization, heart failure, ischemic stroke

## Abstract

**Objective:**

Accumulating evidence suggests that patients with ankylosing spondylitis (AS) have an elevated risk for cardiovascular disease (CVD) and cardiovascular death, however, whether AS has causal effects on the risk of CVD is unclear.Two-sample Mendelian randomization (MR) was utilizedto examine the probable causal link between them.

**Methods:**

Summary statistics from publicly released genome-wide association studies (GWAS) was used to perform MR analyses. Genetically predicted AS was selected as the exposure variable from published GWAS meta-analyses. CVD was adopted as the outcome variable. The inverse variant weighted method was employed to obtain the casual estimates. The robustness of the results was also examined by evaluating the pleiotropy and heterogeneity of single-nucleotide polymorphisms.

**Results:**

According to MR analyses, genetic susceptibility to AS was associated with a high risk of heart failure and ischemic stroke, while negativelygenetic susceptibility was found between AS and peripheral atherosclerosis. No statistical relationship was found between AS and venous thromboembolism, atrial fibrillation, coronary atherosclerosis, and valvular heart disease. Sensitivity analysis showed no evidence of horizontal pleiotropy or heterogeneity.

**Conclusion:**

The present study suggests that AS exerts causal effects on the risk of CVD, including heart failure, ischemic stroke, and peripheral atherosclerosis.

## 1 Introduction

Compared with the general population, patients with ankylosing spondylitis (AS) have a substantially elevatedcardiovascular disease (CVD) risk. In 2009, the European League Against Rheumatism (EULAR) Task Force convened to critically appraise existing evidence on CVD risk in patients with inflammatory joint disorders and determined the enhanced CVD risk in patients with AS (Agca et al., 2017). Their observational studies revealed that AS patients have 1.2–1.4 times increased risk for cardiovascular death compared with the general population (Lehtinen, 1993). Several other studies have reported an increased incidence and prevalence of ischemic heart disease, stroke, and venous thromboembolism in patients with AS (Bremander et al., 2011). With regard to atrial fibrillation and conduction disturbances, some studies have detected an increased rate in patients with AS and reported that the disease often directly affects the conduction system (Lautermann and Braun, 2002).In addition, Siao et al. discovered that patients with AS had a significant risk of valvular heart disease compared to non-AS controls (Siao et al., 2021). Moreover, Ma et al. mentioned that compared to the average population, patients with AS have ahigher chance of valvular heart diseases, conduction disturbances, and cardiomyopathies (Ma et al., 2023).However, these observational studies could be subject to unmeasured confounders, such as type I or II errors, so the true causal effect is difficult to distinguish. To date, causality associations between AS and CVD outcomes remain uncertain.

Mendelian randomization (MR) is a well-acknowledged method to assess the causal inference of an exposure in an outcome by using genetic variants as instrumental variables (IVs) (Smith and Ebrahim, 2003). Since genetic variants are fixed regardless of the development or progression of the disease, this method is able to avoid reverse causality. Single-nucleotide polymorphisms (SNPs) have beendeterminedby genome-wide association studies (GWAS) to be robustly associated with an exposure; they are independent genetic predictors and have been treated as IVs. Under a number of assumptions, MR analysis can yield an exposure–outcome relation that isunlikely to be biased by unobserved confounders. In this study, MR analysis was applied to determine if anunderlying causal relationship of genetic susceptibilityexists between AS and different types of CVD, with the exception of mediation by other factors.

## 2 Methods

### 2.1 Study design overview

Exposure data were derived from a large meta-analysis of GWAS for AS that included 1,462 cases and 164,682 controls. Concerning the outcome dataset, GWAS data for venous thromboembolism were obtained from FinnGen (finn-b-I9_VTE) and included 9,176 cases and 209,616 controls. The outcome dataset for heart failure was derived from the European Bioinformatics Institute database and included 47,309 cases and 930,014 controls (ebi-a-GCST009541). The outcome dataset for atrial fibrillation were derived from five cohort studies and included 60,620 cases and 970,216 controls (ebi-a-GCST006414). SNPs for ischemic stroke were retrieved from a public GWAS meta-analysis and included 34,217 cases and 406,111 controls (ebi-a-GCST005843). The summary data on peripheral atherosclerosis and coronary atherosclerosis were derived from a GWAS that included 6,631 cases and 162,201 controls (finn-b-DM_PERIPHATHERO) and a GWAS that included 14,334 cases and 346,860 controls (ukb-d-I9_CORATHER), respectively. GWAS data for valvular heart disease were obtained from FinnGen (finn-b-I9_VHD) and included 38,209 cases and 156,711 controls.The demographic characteristicsincluded in this study are listed in Table 1.

**TABLE 1:** Data sources and instrumental variables strength assessment.

Traits	Data sources	Sample size (cases/control)	Ancestry	*R* ^2^ (%) for AS (Total)	F for AS (Total)
**Exposure**					
Ankylosing Spondylitis	finn-b-M13_ANKYLOSPON	1,462/164,682	European		
**Outcomes**					
Venous thromboembolism	finn-b-I9_VTE	9,176/209,616	European	0.009	4618.80
Heart failure	ebi-a-GCST009541	47,309/930,014	European	0.0035	177.84
Atrial fibrillation	ebi-a-GCST006414	60,620/970,216	European	0.0074	3685.71
Ischemic stroke	ebi-a-GCST005843	34,217/406,111	European	0.001	490.81
Peripheral atherosclerosis	finn-b-DM_PERIPHATHERO	6,631/162,201	European	0.001	490.80
Coronary atherosclerosis	ukb-d-I9_CORATHER	14,334/346,860	European	0.0012	624.69
Valvular heart disease including rheumatic fever	finn-b-I9_VHD	38,209/156,711	European	0.0032	1625.87

**FIGURE 1:**
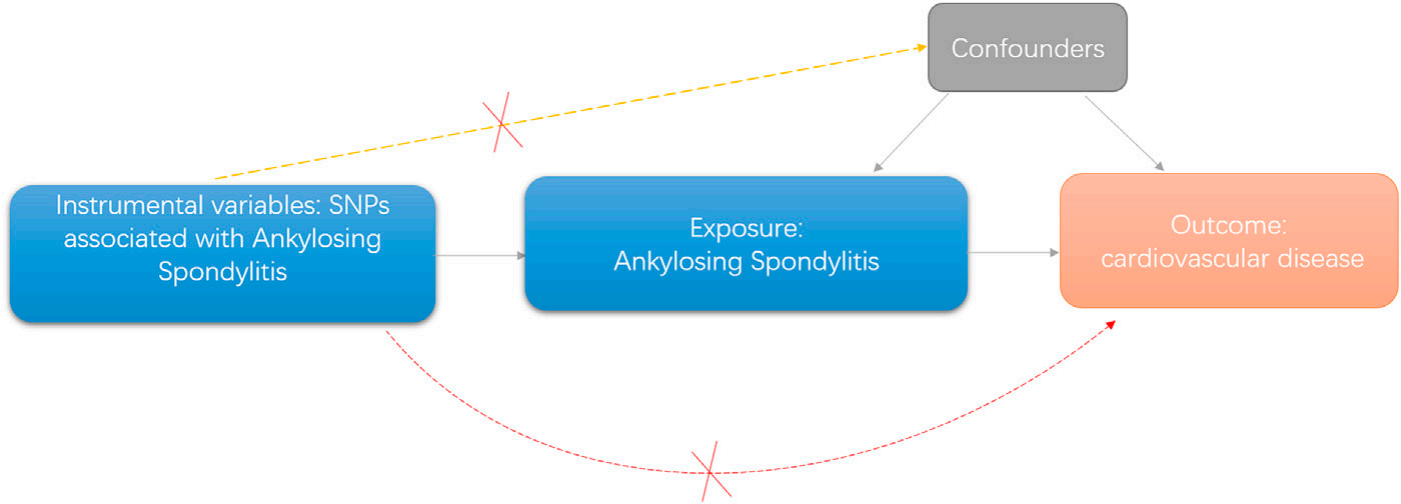
Diagram of two-sample Mendelian randomization analysis of present study.

A directed acyclic graph was used to assess the causal effect between exposures and outcomes (Figure 1). To obtain reliable results, we used genetic variants that conformed to three principles, namely, 1) genetic variants that were associated with the exposure, 2) genetic variants that were not related to any confounders of the exposure–outcome association, and 3) genetic variants that exerted effects on the outcome only via exposure (Bowden and Holmes, 2019).

### 2.2 Selection of IVs

SNPs associated withassociated with AS were selected as IVs (*p* < 5 × 10^−8^). The IVs (*r* ^2^ < 0.01, windows size = 10,000 kb) were clumped to remove the SNPs with strong linkage disequilibrium, since these may cause biased results. Moreover, PhenoScanner V2 was searched to exclude potential pleiotropic effects (Kamat et al., 2019). We excluded the SNPs that related to the outcomes.

To overcome weak-tool bias, variance (*R* ^2^) and F statistics were appliedto evaluate the strength of the IVs(Burgess and Thompson, 2011; Bowden et al., 2016b). The calculation of the F statistic was as follow:

$$F=\frac{N-K-1}{K}\times \frac{R2}{1-R2}$$

*R* ^2^ refers to the cumulative explained variance of the selected SNP during exposure. *K* represents the number of SNPs for the final analysis. *N* is the number of samples (size) of the selected GWAS. F > 10 meansweak-tool bias could be avoided in the results of the MR analysis.

### 2.3 Two-sample MR analysis

We employed two-sample MR methods to identify the causative effect between AS and CVD.The main analytical method was the inverse variance-weighted method (IVW) (Bowden et al., 2016a). Meanwhile, MR-Egger regression, weighted median, weighted mode, and simple mode were also used to validate the results. (Bowden et al., 2015; Nguyen et al., 2015; Bowden et al., 2016a). AS was regarded as exposure, and CVD was regarded as an outcome. Odds ratio (OR) and 95% confidence intervals (CIs) of CVD was used to estimated the effect of AS on CVD.MR–Egger regression was applied to identify the horizontal pleiotropy pathway between the IVs and the outcome (Bowden et al., 2015). MR-pleiotropy residual sum and outlier (MR-PRESSO) was used to infer the causal relationship (Verbanck et al., 2018).The overall pleiotropy was detected by MR-PRESSO goal test.The SNPs with *p* < 0.05 were removed as outlier instruments. The process was repeated until the global test was not significant (*p* > 0.05) (Yuan et al., 2020).

### 2.4 Sensitivity analyses

We applied various methods for sensitivity analyses in this study. First, the heterogeneity among the estimates from each SNP was assessed by Cochran’s Q test. The Q test can also help select an appropriate analysis method. A *p*-value greater than 0.05 indicatedthatthereisnoheterogeneityexisting, therefore, the main method would be the fixed-effects IVW method. When the *p*-value is less than or equal to 0.05, the random-effects model was applied.Second, a leave-one-out sensitivity analysis was conducted by removing each SNP in turn and employing the IVW method. The effect of the remaining SNPs was used to evaluate the stability of the effect sizes and identify the individual SNP that influenced the association disproportionately (Harroud et al., 2021).Third, the MR–Egger intercept method was used to test the horizontal pleiotropy of the IVs. *p* < 0.05 meant that the IVW estimate might be biased. Last, funnel and forest plots were used to evaluated pleiotropy.

All statistical analyseswere did by R software (version 4.0.5, R Foundation for Statistical Computing, Vienna, Austria), and the two-sample MR and MR-PRESSO packages in the software were applied. *p* < 0.05 was considered statistically significant. All *p* values were two-sided.

## 3 Results

### 3.1 Genetic variant selection of SNPs and CVD outcomes

The IVs that were significantly related to AS (*p* < 5 × 10^−8^) and the removed LD (*R* ^2^ < 0.001, 10,000 kb) were extracted from GWAS. Subsequently, SNPs related to CVD were searched in the PhenoScanner database. The screened SNPs were included in the analyses (Supplementary Tables S1–S6). We excluded one SNP, rs13033284, from the analysis of the causal relationship between AS and peripheral atherosclerosis. No evidence of weak-tool bias was found in the IV strength test (F statistic >10). The F and *R* ^2^ values are listed in Table 1.

### 3.2 Causal association between AS and CVD outcomes

**FIGURE 2:**
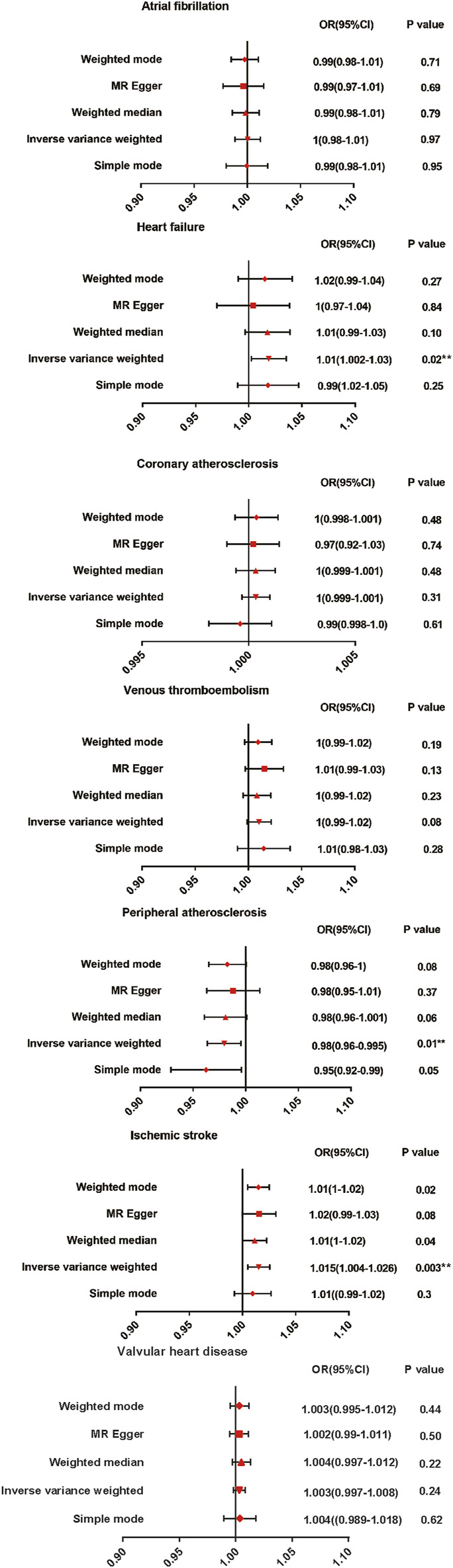
Mendelian randomization estimates of AS on the risk for CVD. OR, Odds ratio; CI, Confidence interval.

The results are shown in Figure 2. The IVW method indicated that AS was associated with a high risk of heart failure and ischemic stroke. Compared with the control patients, the patients with AS had a 1.01-fold risk of heart failure (OR = 1.010%, 95% CI = 1.002–1.030, *p* = 0.020) and a 1.015-fold risk of ischemic stroke (OR = 1.015%, 95% CI = 1.004–1.026, *p* = 0.003). AS had a negative effect on the risk of peripheral atherosclerosis, with a 1 SD increase in AS. The risk of peripheral atherosclerosis decreased by approximately 2% (OR = 0.980%, 95% CI = 0.96–0.995, *p* = 0.010) according to the IVW method. No significant difference was observed in the prevalence of atrial fibrillation (OR = 1.000%, 95% CI = 0.980–1.010, *p* = 0.970), coronary atherosclerosis (OR = 1.000%, 95% CI = 0.999–1.001, *p* = 0.310), venous thromboembolism (OR = 1.000%, 95% CI = 0.990–1.020, *p* = 0.08), andvalvular heart disease (OR = 1.003%, 95% CI = 0.998–1.008, *p* = 0.237)between the patients with AS and the controls. A scatter plot is shown in Supplementary Figure S1.

### 3.3 MR sensitivity analyses

Since the *p* values were all greater than 0.05, there is no heterogeneities (Table 2). Therefore, the fixed-effect IVW method was used as the main analytical method in this study. Moreover, limited evidence of pleiotropy in the IVs of AS with any CVD was displayed from the MR–Egger regression intercept. In addition, according to the leave-one-out test, the MR results were not significantly affected by a single SNP leave-out (Supplementary Figure S2).Forest and funnel plots are given in Supplementary Figures S3, S4 to intuitively show heterogeneity.

**TABLE 2:** Pleiotropy and heterogeneity test of the AS IVs from CVD GWAS.

Outcome	Pleiotropy test			Heterogeneity test					
	MR-Egger			MR-Egger			Inverse-variance weighted		
	Intercept	SE	P	Q	Q_df	Q_pval	Q	Q_df	Q_pval
Venous thromboembolism	0.014	0.008984	0.129899	11.79196	11	0.379482	12.3267	12	0.419811
Heart failure	0.0035	0.0173	0.844707	1.457358	7	0.983749	2.421306	8	0.9653
Atrial fibrillation	−0.00388	0.009725	0.69	18.98599	10	0.05	19.56	11	0.06
Ischemic stroke	0.015369	0.0079	0.083587	14.04531	9	0.120723	14.05092	10	0.170682
Peripheral atherosclerosis	−0.01219	0.013012	0.370821	13.06898	10	0.21984	14.02458	11	0.231636
Coronary atherosclerosis	0.000208	0.000629	0.748794	9.07647	8	0.335886	9.146116	9	0.423897
Valvular heart disease including rheumatic fever	0.0049	0.0043	0.2202	42.53878	38	0.281944	42.54266	39	0.321105

## 4 Discussion

Concerning AS, articular manifestations are usually been focused and treated carefully. However, extra-articular manifestations are easily neglected.Compared to the average population, patients with AS have ahigher chance of CVD (Ma et al., 2023).The mortality rate for AS patientswith CVD is estimated to be 2 times higher thanthat for the normal population (El Maghraoui, 2011). Therefore, it is of great significance to study the causal relationship between AS and CVD to guide the future treatment.In this study, MR analysis was used to systematically determine the potential causal relationship between AS susceptibility and CVD risk. According to the results, genetic liability to AS was positively associated with heart failure and ischemic stroke and negatively associated with peripheral atherosclerosis. To our knowledge, this MR study is the first to focus on the potential causal link between AS and CVD.

Previous studies discovered that AS is associated with increased incidence of circulatory system diseases (Papagoras et al., 2020). Systemic rheumatic inflammation would mediate these associations and disease itselfmay be independently (Papagoras et al., 2013; Gao et al., 2022). However, an analysis of a large database in Israel showed that patients with AS have a high prevalence of CVD, but after adjusting for traditional risk factors, this excess prevalence becomes insignificant (Vinker Shuster et al., 2018). Therefore, with the help of MR analyses, the valid causal association between AS and CVD can be determined.

Comparing general population, cardiovascular disease increases mortality by approximately twofold in patients with AS (Szabo et al., 2011). A meta-analysis found an increased risk of impaired left ventricular function and left-sided heart failure in AS (Heslinga et al., 2014; Braun et al., 2017). A nationwide longitudinal cohort study in Korea also discovered that the incidence rates of congestive heart failure and death are increased in patients with AS (Bae et al., 2019). These results are in accordance with ours. We found a positive causal relationship between AS and heart failure. The mechanism through which heart failure increases in patients with AS is still uncertain. Inflammation and amyloidosis in AS may cause fibrosis in the aortic root and thickening in the adjacent ventricular septum, which may be the reason for heart failure in AS (Ha et al., 2009). Heart structural changes, muscle remodeling (includingapoptosis, increased left ventricular mass, diastolic dysfunction and myocardial inflammation/fibrosis) and functional abnormalities (biochemical and ionchannel remodeling) may be originated frominflammatory arthritis including AS (Castaneda et al., 2018).In addition, Secondary amyloidosis is an important complicationof chronic inflammatory diseases andAS are known to be associated with the developmentof amyloidosis (Dönmez et al., 2013).Our study offers an explanation of gene susceptibility to heart failure in patients with AS.

Moreover, our study found a causal association between AS and ischemic stroke. The findings of the majority of previous case-control and observational studies on the association between AS and stroke are controversial (Eriksson et al., 2017). Lee et al., 2018 conducted a large cohort study and discovered that patients with AS have a statistically significant increase in ischemic stroke risk relative to patients without AS. While, a retrospective cohort study found no link between AS and stroke (Brophy et al., 2012). However, in Brophy’s study, possible confounding factors, such as body mass index and lipoprotein cholesterol, should be noted. One of the most important characteristics of patients with AS is the elevation of inflammatory markers, which may result in thickened intima and media blood arteries (Behari et al., 2018). In addition, the disproportionate frequency of methylenetetrahydrofolate reductase (C677T) gene polymorphism in patients withAS may also be a potential etiopathological factor for the development of ischemic stroke (Behari et al., 2018). The association between the C677T of methylenetetrahydrofolate reductase and the risk for ischemic stroke has been studied in different populations. Many studies haveshown that the polymorphism is a risk factor for ischemic stroke (Zhu et al., 2015).Our MR analysis strongly supports the role of AS in the risk of ischemic stroke and indicates that individuals with AS may benefit from stroke prevention treatments.

Our results are inconsistent with those of many previous studies in terms of the association between AS and peripheral atherosclerosis risk. We found a negative causal association between AS and peripheral atherosclerosis. The majority of studies reported that AS is associated with accelerated atherosclerosis and enhanced cardiovascular morbidity and mortality (Łosińska et al., 2019). However, no Grade A evidence links atherosclerosis to AS because most studies are cross sectional and involve small patient samples and controls.Platelets have beenrecognized as crucial regulators of inflammatory processes undervarious pathophysiological conditions.Previous study suggested that platelet may contribute to the inflammationseverity and treatment outcomes in AS patients (Qian et al., 2020).Stephan Zeibigreported that platelets contribute to increased tissueoxidized low-density lipoprotein in the aortic wall but not in peripheral blood, which may be the evidence of the negative causal association between AS and peripheral atherosclerosis (Zeibig et al., 2019). Since platelets may not influence the level of oxidized low-density lipoprotein in peripheral blood, the incidence of peripheral atherosclerosis in AS patients may not elevated.Moreover, Hou et al. found that the number of CD68+/RANK + cells is increased in peripheral blood from patients with AS (Hou et al., 2018). CD68 can be considerably upregulated in macrophages responding to inflammatory stimuli. Song et al., 2011 reported that CD68-deficient mononuclear phagocytes show a potent lipid uptake, indicating that the elevation of CD68 may contribute to antiatherosclerosis (Chistiakov et al., 2017). This result may explain the potential protective effect of AS on the risk of peripheral atherosclerosis.

The main advantage of this study is the implementation of the MR approach to determine causal associations. MR analysis diminishes the interference of confounding factors, and studies that use MR analysis might be more convincing than observational studies. To our knowledge, the study is the first MR analysis concerning this topic. However, some limitations should be carefully discussed in interpretingthe results. First, the present study was based on publicly available summary-level data, and the provided data prevented us from performing other subgroup analyses to address associations with study-specific factors. Second, our study focused on the effect of AS on the occurrence of CVD. Therefore, a precisely designed bidirectional two-sample MR study is necessary to verify the effect of CVD on AS. Last, because this study was limited to participants of European descent, population confinement might limit the generalizability of the results to other populations.

In conclusion, our study demonstrated the causal association between AS and increased risks of heart failure and ischemic stroke and a decreased risk of peripheral atherosclerosis. Our research can help us to understand the basic disease mechanisms of AS and offer comprehensive CVD assessments and treatments for patients with AS.

## Data Availability

The original contributions presented in the study are included in the article/Supplementary Material, further inquiries can be directed to the corresponding author.
