# Supplementary material for: Effects of ankylosing spondylitis on cardiovascular disease: aMendelian randomization study
Source: Front Genet. 2024 Jun 26;15:1359829. doi: 10.3389/fgene.2024.1359829 (PMC11233707; doi:10.3389/fgene.2024.1359829)
Supplement: Supplementary file 1 [file DataSheet1.pdf]

Supplementary Table 1 Mendelian randomization analysis of Ankylosing Spondylitis and Atrial fibrillation

SNP	A1	A2	Chr	Position	Ankylosing Spondylitis			Atrial fibrillation		
					Beta	SE	P_val	Beta	SE	P_val
rs10807943	C	T	7	5340664	-0.5628	0.0812	4.10E-12	0.0198	0.0125	0.1146
rs112733823	T	C	6	30775277	0.36	0.0468	1.43E-14	0.0443	0.0179	0.01309
rs13033284	C	T	2	62581573	-0.2214	0.0386	9.67E-09	-0.0015	0.0069	0.8256
rs16894011	A	T	6	28332453	2.1075	0.0886	5.32E-125	-0.0236	0.0162	0.1464
rs181316459	C	G	7	5473610	0.9847	0.1004	1.02E-22	-0.0027	0.0501	0.957
rs34982906	C	T	6	24071028	0.8211	0.0918	3.82E-19	0.0466	0.0205	0.02263
rs62394289	A	G	6	25848911	0.3738	0.0557	1.90E-11	0.0101	0.0103	0.3298
rs76644067	A	G	6	35741951	0.7328	0.0946	9.44E-15	-0.0102	0.0172	0.5561
rs79693223	T	C	6	33901952	1.2953	0.1062	3.36E-34	0.004	0.0187	0.8314
rs9264277	C	T	6	31224667	0.5198	0.0448	3.73E-31	-0.0098	0.0107	0.3637
rs9265893	A	G	6	31313339	1.9426	0.0588	1.00E-200	-0.0106	0.0105	0.3113
rs9265893	C	G	6	31313339	1.9426	0.0588	1.00E-200	-1.00E-04	0.0116	0.9941

rs9378220	A	C	6	30077135	-0.6933	0.0558	1.97E-35	-0.005	0.0099	0.6131
rs9391773	T	G	6	31318585	2.6349	0.0702	1.00E-200	0.028	0.0263	0.287

Supplementary Table 2 Mendelian randomization analysis of Ankylosing Spondylitis and Coronary atherosclerosis

SNP	A1	A2	Chr	Position	Ankylosing Spondylitis			Coronary atherosclerosis		
					Beta	SE	P_val	Beta	SE	P_val
rs10807943	C	T	7	5340664	-0.5628	0.0812	4.10E-12	0.000247	0.000723	0.732248
rs112733823	T	C	6	30775277	0.36	0.0468	1.43E-14	5.68E-05	0.000701	0.935478
rs13033284	C	T	2	62581573	-0.2214	0.0386	9.67E-09	-0.00057	0.000465	0.217327
rs16894011	A	T	6	28332453	2.1075	0.0886	5.32E-125	0.001336	0.001197	0.264259
rs34982906	C	T	6	24071028	0.8211	0.0918	3.82E-19	0.002301	0.001235	0.06243
rs62394289	A	G	6	25848911	0.3738	0.0557	1.90E-11	-0.00041	0.000704	0.562201
rs76644067	A	G	6	35741951	0.7328	0.0946	9.44E-15	-0.00049	0.001168	0.672009
rs79693223	T	C	6	33901952	1.2953	0.1062	3.36E-34	-0.00018	0.001159	0.878235
rs9264277	C	T	6	31224667	0.5198	0.0448	3.73E-31	0.000641	0.00047	0.172263
rs9265893	A	G	6	31313339	1.9426	0.0588	1.00E-200	0.00082	0.000582	0.158707
rs9378220	A	C	6	30077135	-0.6933	0.0558	1.97E-35	0.000746	0.00063	0.23637

Supplementary Table 3 Mendelian randomization analysis of Ankylosing Spondylitis and Heart failure

SNP	A1	A2	Chr	Position	Ankylosing Spondylitis			Heart failure		
					Beta	SE	P_val	Beta	SE	P_val
rs10807943	C	T	7	5340664	-0.5628	0.0812	4.10E-12	-0.0147	0.0159	0.354
rs112733823	T	C	6	30775277	0.36	0.0468	1.43E-14	0.0036	0.02	0.8584
rs13033284	C	T	2	62581573	-0.2214	0.0386	9.67E-09	-0.0123	0.0081	0.1268
rs16894011	A	T	6	28332453	2.1075	0.0886	5.32E-125	0.0339	0.0188	0.070659
rs34982906	C	T	6	24071028	0.8211	0.0918	3.82E-19	0.0215	0.0235	0.3604
rs62394289	A	G	6	25848911	0.3738	0.0557	1.90E-11	0.0148	0.0121	0.2237
rs76644067	A	G	6	35741951	0.7328	0.0946	9.44E-15	0.0151	0.0206	0.4634
rs79693223	T	C	6	33901952	1.2953	0.1062	3.36E-34	0.0234	0.0216	0.278
rs9264277	C	T	6	31224667	0.5198	0.0448	3.73E-31	0.0053	0.0138	0.7036
rs9265893	A	G	6	31313339	1.9426	0.0588	1.00E-200	-0.0031	0.013	0.8149
rs9378220	A	C	6	30077135	-0.6933	0.0558	1.97E-35	-0.0038	0.0108	0.7229

Supplementary Table 4 Mendelian randomization analysis of Ankylosing Spondylitis and Ischemic stroke

SNP	A1	A2	Chr	Position	Ankylosing Spondylitis			Ischemic stroke		
					Beta	SE	P_val	Beta	SE	P_val
rs10807943	C	T	7	5340664	-0.5628	0.0812	4.10E-12	-0.0024	0.0154	0.8745
rs112733823	T	C	6	30775277	0.36	0.0468	1.43E-14	0.0121	0.0173	0.4853
rs13033284	C	T	2	62581573	-0.2214	0.0386	9.67E-09	0.0023	0.0084	0.788099
rs16894011	A	T	6	28332453	2.1075	0.0886	5.32E-125	0.0176	0.0158	0.2668
rs181316459	C	G	7	5473610	0.9847	0.1004	1.02E-22	-0.0013	0.0203	0.948
rs34982906	C	T	6	24071028	0.8211	0.0918	3.82E-19	-0.0427	0.0318	0.1795
rs62394289	A	G	6	25848911	0.3738	0.0557	1.90E-11	0.0431	0.0144	0.002714
rs76644067	A	G	6	35741951	0.7328	0.0946	9.44E-15	0.0314	0.0211	0.1367
rs79693223	T	C	6	33901952	1.2953	0.1062	3.36E-34	0.0274	0.0236	0.2455
rs9264277	C	T	6	31224667	0.5198	0.0448	3.73E-31	-0.0023	0.0108	0.8328
rs9265893	A	G	6	31313339	1.9426	0.0588	1.00E-200	0.0033	0.0137	0.8095
rs9378220	A	C	6	30077135	-0.6933	0.0558	1.97E-35	-0.0064	0.014	0.6494

rs9391773	T	G	6	31318585	2.6349	0.0702	1.00E-200	0.0547	0.0177	0.001989

Supplementary Table 5 Mendelian randomization analysis of Ankylosing Spondylitis and Peripheral atherosclerosis

SNP	A1	A2	Chr	Position	Ankylosing Spondylitis			Peripheral atherosclerosis		
					Beta	SE	P_val	Beta	SE	P_val
rs10807943	C	T	7	5340664	-0.5628	0.0812	4.10E-12	-0.0176	0.0421	0.676299
rs112733823	T	C	6	30775277	0.36	0.0468	1.43E-14	-0.0176	0.0248	0.4762
rs13033284	C	T	2	62581573	-0.2214	0.0386	9.67E-09	0.0443	0.021	0.03512
rs16894011	A	T	6	28332453	2.1075	0.0886	5.32E-125	-0.1124	0.0392	0.004161
rs181316459	C	G	7	5473610	0.9847	0.1004	1.02E-22	-0.0426	0.049	0.3846
rs34982906	C	T	6	24071028	0.8211	0.0918	3.82E-19	0.0625	0.0454	0.1691
rs62394289	A	G	6	25848911	0.3738	0.0557	1.90E-11	0.0035	0.0298	0.9054
rs76644067	A	G	6	35741951	0.7328	0.0946	9.44E-15	-0.0445	0.0475	0.3495
rs79693223	T	C	6	33901952	1.2953	0.1062	3.36E-34	-0.049	0.0498	0.325
rs9265893	A	G	6	31313339	1.9426	0.0588	1.00E-200	0.0805	0.0234	0.000587
rs9265893	C	G	6	31313339	1.9426	0.0588	1.00E-200	-0.0536	0.0266	0.0441
rs9378220	A	C	6	30077135	-0.6933	0.0558	1.97E-35	9.00E-04	0.0288	0.9743

rs9391773	T	G	6	31318585	2.6349	0.0702	1.00E-200	-0.0089	0.0304	0.769501
rs9391773	T	C	6	31318585	2.6349	0.0702	1.00E-200	-0.1038	0.0635	0.1022

Supplementary Table 6 Mendelian randomization analysis of Ankylosing Spondylitis and Venous thromboembolism

SNP	A1	A2	Chr	Position	Ankylosing Spondylitis			Venous thromboembolism		
					Beta	SE	P_val	Beta	SE	P_val
rs10807943	C	T	7	5340664	-0.5628	0.0812	4.10E-12	-0.0075	0.0328	0.8193
rs112733823	T	C	6	30775277	0.36	0.0468	1.43E-14	0.0139	0.0193	0.4717
rs13033284	C	T	2	62581573	-0.2214	0.0386	9.67E-09	0.0178	0.0164	0.2781
rs16894011	A	T	6	28332453	2.1075	0.0886	5.32E-125	0.0373	0.0304	0.2205
rs181316459	C	G	7	5473610	0.9847	0.1004	1.02E-22	-0.0319	0.0387	0.4101
rs34982906	C	T	6	24071028	0.8211	0.0918	3.82E-19	-0.0248	0.0356	0.4873
rs62394289	A	G	6	25848911	0.3738	0.0557	1.90E-11	-0.017	0.023	0.459501
rs76644067	A	G	6	35741951	0.7328	0.0946	9.44E-15	0.0461	0.0373	0.2167
rs79693223	T	C	6	33901952	1.2953	0.1062	3.36E-34	0.0702	0.0391	0.072671
rs9264277	C	T	6	31224667	0.5198	0.0448	3.73E-31	-0.0156	0.018	0.3864
rs9265893	C	G	6	31313339	1.9426	0.0588	1.00E-200	0.0113	0.0208	0.5871
rs9265893	A	G	6	31313339	1.9426	0.0588	1.00E-200	-0.0369	0.0183	0.04435

rs9378220	A	C	6	30077135	-0.6933	0.0558	1.97E-35	-0.0436	0.0224	0.05169
rs9391773	T	G	6	31318585	2.6349	0.0702	1.00E-200	0.0218	0.0236	0.3548
rs9391773	T	C	6	31318585	2.6349	0.0702	1.00E-200	0.052	0.0495	0.2935

Supplementary Table 7 Mendelian randomization analysis of Ankylosing Spondylitis and Valvular heart disease

SNP	A1	A2	Chr	Position	Ankylosing Spondylitis			Venous thromboembolism		
					Beta	SE	P_val	Beta	SE	P_val
rs115490327	A	A	6	32695647	1.4927	0.182	2.33E-16	0.004	0.0392	0.9186
rs115537782	A	A	6	27907372	1.9981	0.1356	3.98E-49	0.0083	0.0271	0.7592
rs12195837	T	T	6	25434243	0.4224	0.0634	2.62E-11	0.0087	0.0151	0.5659
rs12200941	G	G	6	34420570	0.2553	0.0444	8.63E-09	-0.0028	0.0108	0.7941
rs12209273	C	C	6	33849073	1.1024	0.0772	3.07E-46	0.0084	0.0166	0.6125
rs12664075	A	A	6	30354706	1.0413	0.0753	1.70E-43	0.0015	0.0164	0.9279
rs13033284	C	C	2	62581573	-0.2214	0.0386	9.67E-09	-0.0152	0.0094	0.1073
rs142526981	A	A	6	36997080	0.8225	0.1391	3.36E-09	0.0429	0.0316	0.1739
rs147086576	A	A	6	26847011	0.9433	0.0734	7.75E-38	-0.0092	0.0161	0.5707
rs1511478	T	T	6	23976276	0.4126	0.0685	1.72E-09	-0.0258	0.0162	0.1121
rs1535948	A	A	6	33783883	0.4514	0.0411	5.35E-28	0.0065	0.0099	0.5111
rs16890706	A	A	6	25520094	0.4309	0.0571	4.65E-14	-0.0039	0.0134	0.770801

rs16893801	C	C	6	28174024	2.5818	0.0985	#######	-0.0113	0.0189	0.5487
rs17212937	T	T	6	32681339	0.9952	0.0566	2.96E-69	0.009	0.0125	0.4707
rs181316459	C	C	7	5473610	0.9847	0.1004	1.02E-22	0.0071	0.0221	0.7483
rs2223591	A	A	6	26271241	1.2006	0.1058	7.75E-30	-0.0039	0.0227	0.863
rs2240803	A	A	6	30920957	0.9331	0.0481	6.49E-84	#######	0.0107	0.9755
rs28474889	T	T	6	31575073	0.7638	0.0498	4.54E-53	-0.0212	0.0116	0.06806
rs303888	G	G	6	25035584	-0.2774	0.0385	6.02E-13	-0.0028	0.0094	0.7663
rs3129293	G	G	6	33085010	0.3214	0.0382	4.20E-17	0.0142	0.0093	0.1273
rs34018151	G	G	6	24613120	0.6014	0.0837	6.56E-13	-0.0038	0.0192	0.8447
rs3993757	T	T	6	31590746	2.0555	0.1484	1.22E-43	0.0517	0.0295	0.079299
rs414532	A	A	6	32989858	0.8716	0.0639	2.33E-42	0.0178	0.0143	0.2107
rs4713624	T	T	6	33438220	0.7527	0.0981	1.68E-14	-0.0165	0.0223	0.4586
rs532086	C	C	6	31881309	1.1891	0.0581	5.15E-93	0.0344	0.0126	0.00636
rs55813549	T	T	6	31318646	2.6349	0.0702	#######	0.0169	0.0136	0.2128

rs56073264	A	A	6	26079416	1.0197	0.0696	1.16E-48	0.0047	0.0152	0.757501
rs56220358	T	T	7	5510706	0.28	0.0428	6.29E-11	-0.0131	0.0103	0.204
rs62443225	A	A	7	5482137	0.6277	0.075	5.95E-17	0.0108	0.0173	0.5332
rs6916321	G	G	6	26464789	0.2782	0.0434	1.39E-10	0.0012	0.0106	0.9071
rs6956343	T	T	7	5291400	0.385	0.0535	5.92E-13	-0.0081	0.0127	0.5232
rs6966951	G	G	7	5406057	-0.2415	0.0412	4.62E-09	-0.0097	0.0101	0.3376
rs71555121	A	A	6	24038537	0.2454	0.0434	1.60E-08	0.0089	0.0105	0.3997
rs72833065	C	C	6	24419556	1.119	0.1229	8.78E-20	0.0271	0.0264	0.3043
rs7742085	G	G	6	32746178	0.5857	0.0818	7.82E-13	0.0221	0.0188	0.2404
rs77793619	G	G	6	28192122	1.2307	0.0643	1.27E-81	-0.014	0.0138	0.3101
rs77831243	C	C	6	25170013	1.2037	0.1418	2.06E-17	-0.0144	0.0307	0.6391
rs9266510	C	C	6	31341233	-0.8771	0.0432	1.62E-91	0.0217	0.0101	0.03224
rs9379729	T	T	6	25181627	0.3329	0.0491	1.20E-11	-0.0146	0.0117	0.2132
rs9470177	C	C	6	35912918	0.7368	0.0895	1.85E-16	0.0139	0.0203	0.4942

Supplementary figures

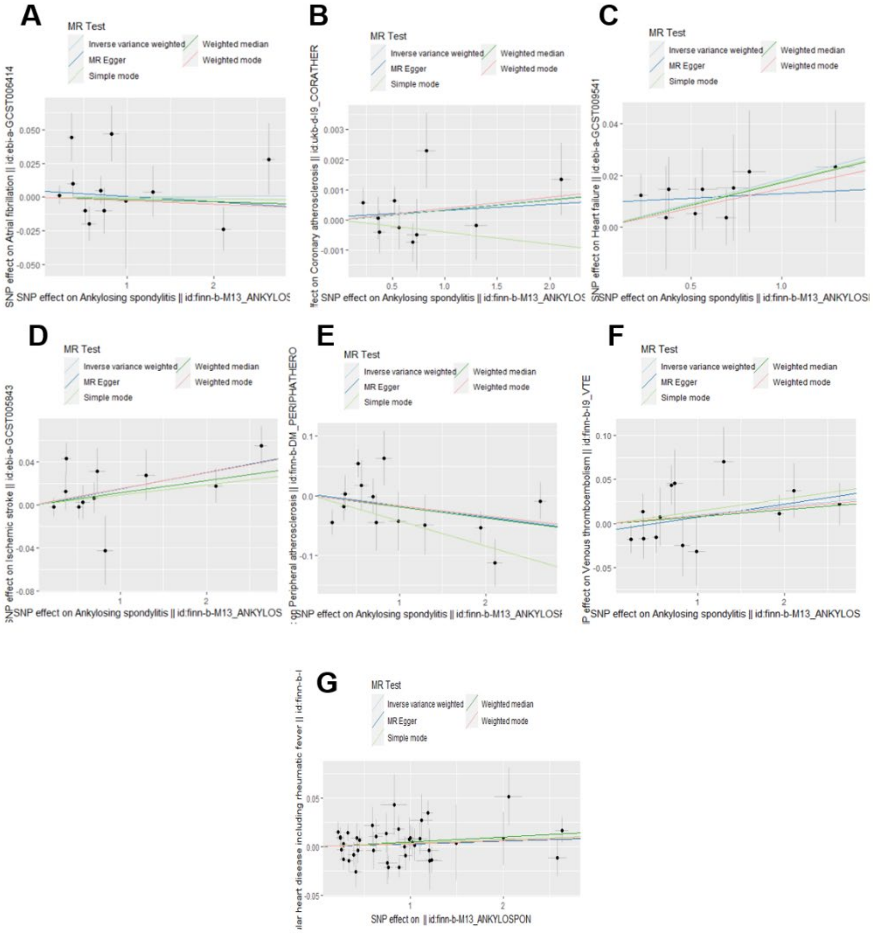

Supplementary Figure 1 Scatter plot: (A) AS and atrial fibrillation; (B) AS and Coronary atherosclerosis; (C) AS and heart failure; (D) AS and Ischemic stroke; (E) AS and Peripheral atherosclerosis; (F) AS and Venous thromboembolism; (G) AS and valvular heart disease

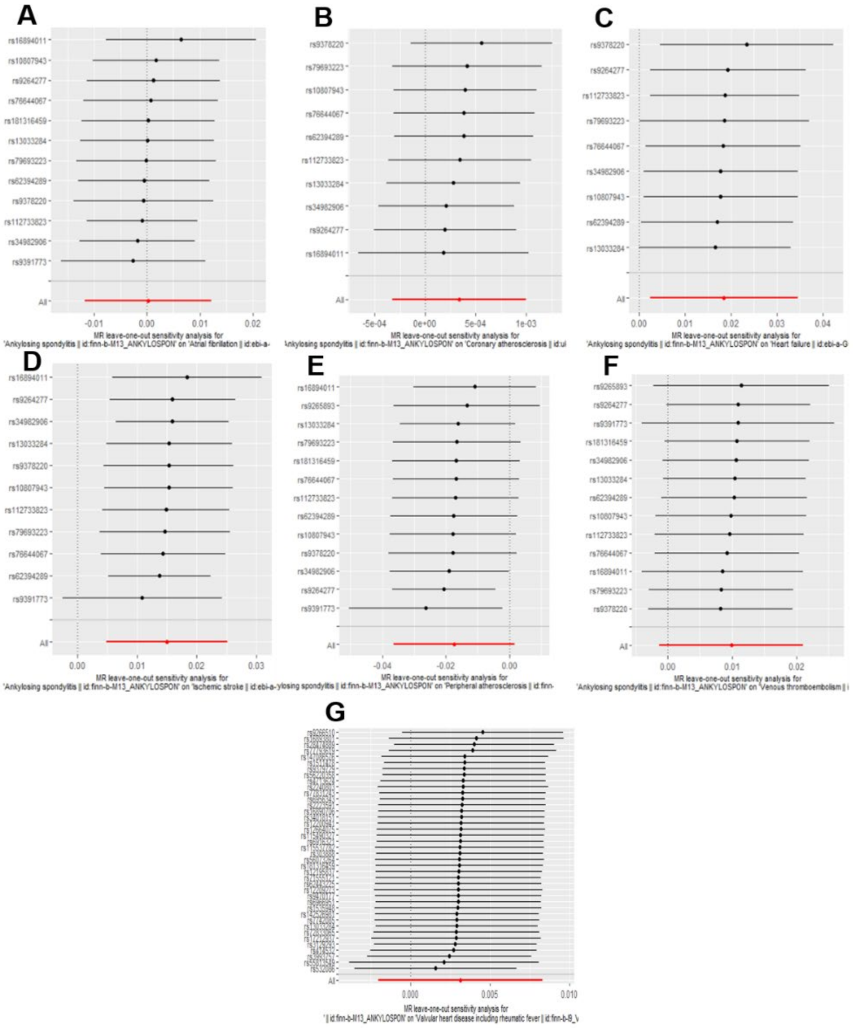

Supplementary Figure 2 Leave-one-out sensitivity analysis: (A) AS and atrial fibrillation; (B) AS and Coronary atherosclerosis; (C) AS and heart failure; (D) AS and Ischemic stroke; (E) AS and Peripheral atherosclerosis; (F) AS and Venous thromboembolism; (G) AS and valvular heart disease

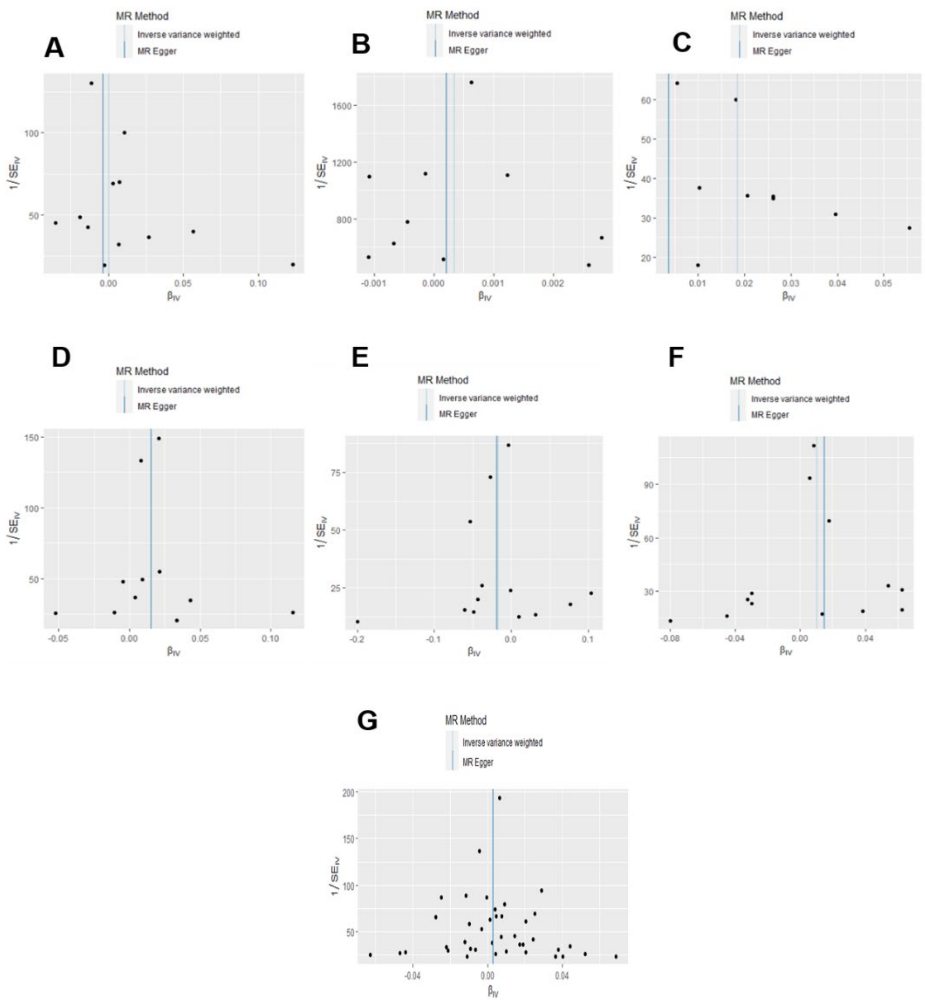

Supplementary Figure 3 Funnel plot: (A) AS and atrial fibrillation; (B) AS and Coronary atherosclerosis; (C) AS and heart failure; (D) AS and Ischemic stroke; (E) AS and Peripheral atherosclerosis; (F) AS and Venous thromboembolism; (G) AS and valvular heart disease

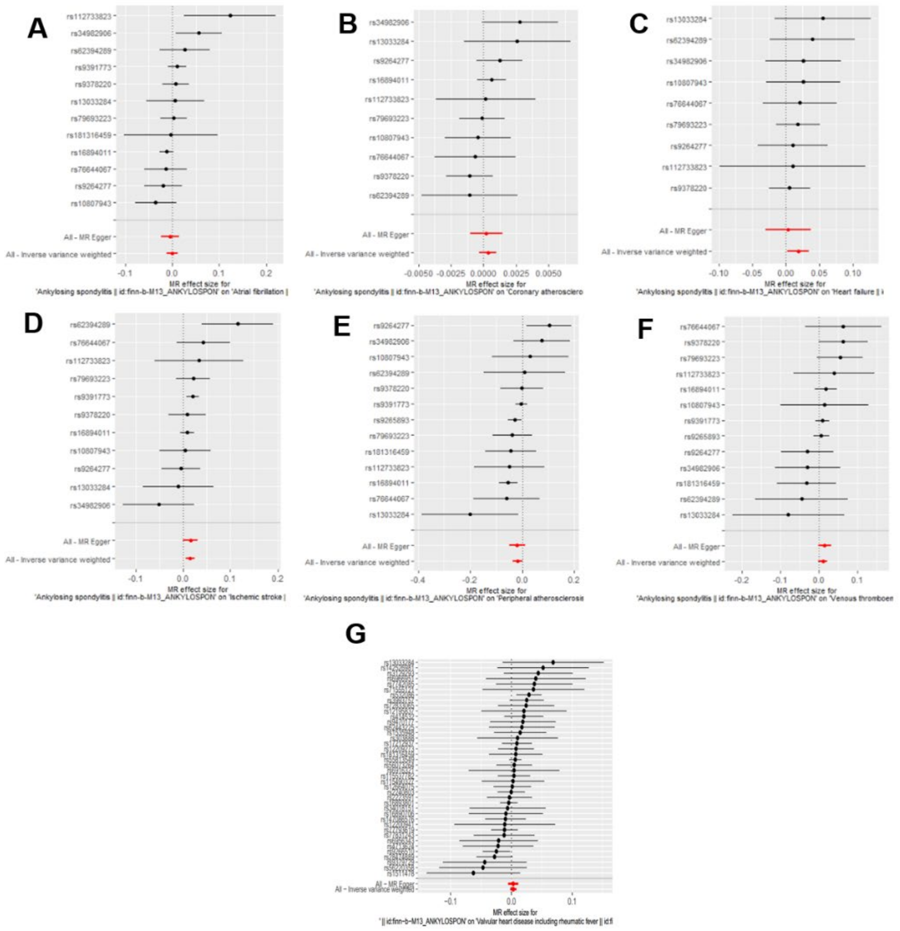

Supplementary Figure 4 Forest plot: (A) AS and atrial fibrillation; (B) AS and Coronary atherosclerosis; (C) AS and heart failure; (D) AS and Ischemic stroke; (E) AS and Peripheral atherosclerosis; (F) AS and Venous thromboembolism; (G) AS and valvular heart disease
